# The Development Prospects and Potential of High Specific Surface Area Materials: A Review of the Use of Porous Framework Materials for the Capture and Filtration of Ammonia

**DOI:** 10.3390/molecules30081737

**Published:** 2025-04-13

**Authors:** Wenhao Yao, Wenying Wu, Yitong Liu, Bingfa Zhu, Jifa Xiao, Teng Zhang, Senliang Xi

**Affiliations:** 1 Key Laboratory of Cluster Science Ministry of Education, Beijing Key Laboratory of Photoelectronic/Electrophotonic Conversion Materials, Advanced Research Institute of Multidisciplinary Science, School of Chemistry and Chemical Engineering, Beijing Institute of Technology, Beijing 100081, China; ywh200012@163.com (W.Y.); wwy15933833065@163.com (W.W.); m18735721812_1@163.com (Y.L.); 2 Advanced Technology Research Institute (Jinan), Beijing Institute of Technology, Jinan 250000, China; zbingfa@163.com (B.Z.); 17662656866@163.com (J.X.)

**Keywords:** ammonia adsorption, porous framework materials, metal–organic frameworks

## Abstract

Ammonia is one of the most widely produced inorganic chemicals, with extensive applications in the military, agricultural, and industrial sectors. However, its strong stimulation and corrosive properties pose significant health risks, as long-term exposure to ammonia environments can lead to respiratory tract damage, loss of consciousness, and even cardiopulmonary dysfunction. Over the years, researchers have focused on exploring suitable materials for ammonia adsorption fields such as activated carbon and zeolites. Porous framework materials (PFMs), including metal–organic frameworks, covalent organic frameworks, and hydrogen-bonded organic frameworks, have emerged as possible ammonia adsorption materials due to their high specific surface area, pore size, and structural adjustability. This review focuses on the research and application of materials with excellent adsorption based on PFMs for ammonia adsorption, highlighting their potential applications and providing insights into future developments in this field.

## 1. Introduction

Since the industrial adoption of the Haber–Bosch process in 1908, ammonia has become one of the most widely used inorganic chemicals, with its annual production exceeding 200 million tons. Ammonia is essential in industries such as petrochemicals, metal manufacturing, paper production, textiles, and agriculture [1,2,3]. However, despite its economic benefits, ammonia emissions pose significant environmental and health risks. NH_3_ is considered an important contributor to the formation of particulate matter 2.5 (PM2.5). Through various physicochemical reactions, particles such as ammonium sulfide and ammonium nitrate form, accounting for approximately 30 wt% of the atmospheric PM2.5 content, with peak levels reaching up to 60 wt% [4,5]. In addition, airborne NH_3_ concentrations above 0.02 vol% can lead to chronic poisoning. Short-term exposure (less than 8 h) to an ammonia atmosphere can irritate the respiratory tract, eyes, and skin, while long-term exposure to NH_3_ concentrations above 300 × 10^−6^ vol% can cause serious illness and even death [6]. Currently, while ammonia adsorption technology at high concentrations is well developed, capturing ammonia molecules at low concentrations remains a critical issue. This is particularly pressing due to the rapid development of the semiconductor industry, where NH_3_ concentrations are strictly limited to 7–345 ppb in semiconductor clean rooms, with less than 2 ppb being permitted in layer presentation/inspection tools, and under 20 ppb in lithographic scanners [7,8,9,10]. Therefore, NH_3_ adsorption at low concentrations is critical for semiconductor production. Despite issues such as equipment corrosion and high operating costs, acid and water scrubbing remain the most common methods for ammonia capture [11]. Porous framework materials (PFMs), especially metal–organic frameworks (MOFs), have gained significant attention over the past two decades due to their high specific surface area, adjustable pore size, and active sites, which can provide metal ions and organic ligands, offering significant potential in the field of gas adsorption [12].

**Figure 1:**
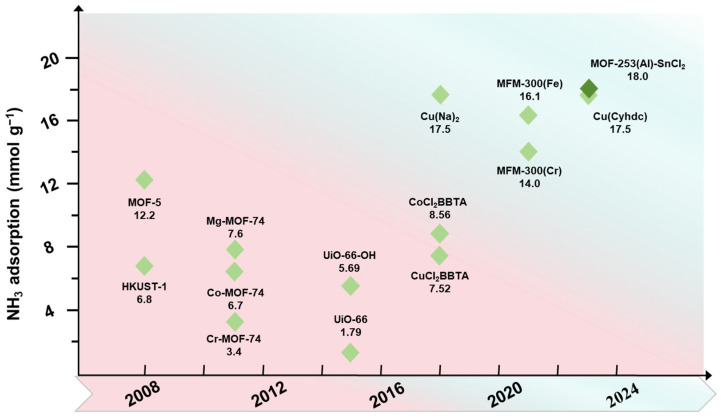
Summary of recent developments in metal–organic frameworks for ammonia adsorption.

In this review, we summarize industrial ammonia abatement techniques and compare the adsorption performances of mature ammonia adsorption materials and PFMs (Figure 1). The prospects for the development of high-performance ammonia adsorbents are also presented.

## 2. Ammonia Filtration Methods

Given the harmful effects of NH_3_ on the environment, capturing ambient ammonia gas is crucial from both an environmental and commercial perspective. Due to ammonia’s extremely high solubility in water (57.1 g L^−1^, 298 K, 1 atm), water scrubbing is the simplest and most common method for adsorbing it. This process involves absorbing NH_3_ using soft water, then distilling the solution to obtain concentrated ammonia water, and then recovering the NH_3_. However, despite its maturity and widespread application, water scrubbing is characterized by high water and energy consumption and a low recovery rate [13]. In contrast, optimized processes such as catalytic oxidation, membrane separation, biological treatment, washing, and adsorption methods offer more efficient and practical alternatives for ammonia capture [14,15,16,17,18,19].

### 2.1. Catalytic Oxidation Process

Catalytic oxidation uses highly selective catalysts to convert NH_3_ into N_2_, NO_x_, and H_2_O [14]. This reaction is considered one of the most significant heterogeneous catalytic processes in industrial and commercial applications. At higher reaction temperatures, NO_x_ is mainly produced, while a mixture of N_2_, N_2_O, and NO forms when the oxidation reaction temperature is below 500 °C. Although catalytic oxidation is a simple, efficient, and stable method, it has a high energy demand and generates NO_x_, which is an exhaust gas that requires additional treatment [15].

The electrochemical oxidation of ammonia is a technique that converts ammonia into hydrazine (N_2_H_4_), nitrite (NO_2_^−^), and nitrate (NO_3_^−^) through the application of Pt-, Ir-, or Ni-based catalysts under an electric field [20]. This process can be conducted under an ambient temperature and pressure, thereby reducing the high energy demand of the traditional catalytic oxidation process. However, under prolonged high-potential operating conditions, the catalyst surface becomes susceptible to oxidative corrosion and irreversible phase transformations, which ultimately leads to the structural collapse of active sites and deterioration of the reaction kinetics. These degradation mechanisms pose critical challenges to the long-term operational stability and catalytic performance of the system [21].

### 2.2. Membrane Separation

Membranes have the advantages of lower maintenance requirements and being easier to install and operate than other traditional technologies. However, despite their significant advantages, membranes still have issues, such as low selectivity for target pollutants under high-throughput conditions, and further development is needed before membranes can be commercially applied [16]. In addition, the working stability of membranes under extreme conditions such as high temperatures, high pressure, and high pollution concentrations requires further study [22].

### 2.3. Biotechnology

Biotechnology mainly includes three NH_3_ treatment methods: biological purifiers, biological filters, and biological drip filters. Biological methods have the advantages of low pressure drops and operating costs; however, the accumulation of biomass and secondary waste remains a significant issue. Furthermore, bacterial discharge poses an environmental risk, further limiting the application of these filters [17].

### 2.4. Washing

Washing mainly relies on scrubbers, which are reactors that are filled with inert or inorganic fillers that have a large surface area and are highly porous. The gas that is to be separated is typically introduced counter-currently, allowing for the gas flow to be in close contact with the water and facilitating the transfer of NH_3_ from the gas phase to the liquid phase. In this process, the mass transfer rate is directly proportional to the NH_3_ concentration gradient between the gas and liquid phases [23]. Therefore, diluted sulfuric acid or phosphoric acid solutions are typically added to the water to maintain a pH below 4, thereby enhancing the mass transfer rate [24]. This method can withstand a wide temperature range, with the help of a robust scrubber. However, similarly to biological treatment methods, the scrubbing method faces high operational costs due to the need for extensive maintenance as a result of corrosion and structural degradation [18].

### 2.5. Adsorption

Among the various methods for NH_3_ purification, adsorption has gained attention and is of significant practical interest. Adsorption can be categorized into physisorption and chemisorption, each with distinct removal mechanisms. Physisorption is mainly driven by van der Waals forces between molecules, while chemisorption involves adsorption behavior that is driven by chemical bonding on the substance surface. Activated carbon (AC), zeolites, and metal oxides have been widely used as adsorption materials in recent years [19]. MOFs have received extensive attention over the past two decades due to their beneficial properties, such as a high specific surface area, large pore volume, and adjustable function, making them potential tools for NH_3_ adsorption and separation under different conditions [25].

In summary, although these technologies have proven to be viable ammonia abatement methods, there are technical and economic drawbacks in practical applications (Table 1). This facilitates the development of enhanced ammonia adsorption approaches.

## 3. Commonly Used Ammonia Adsorbents

**Figure 2:**
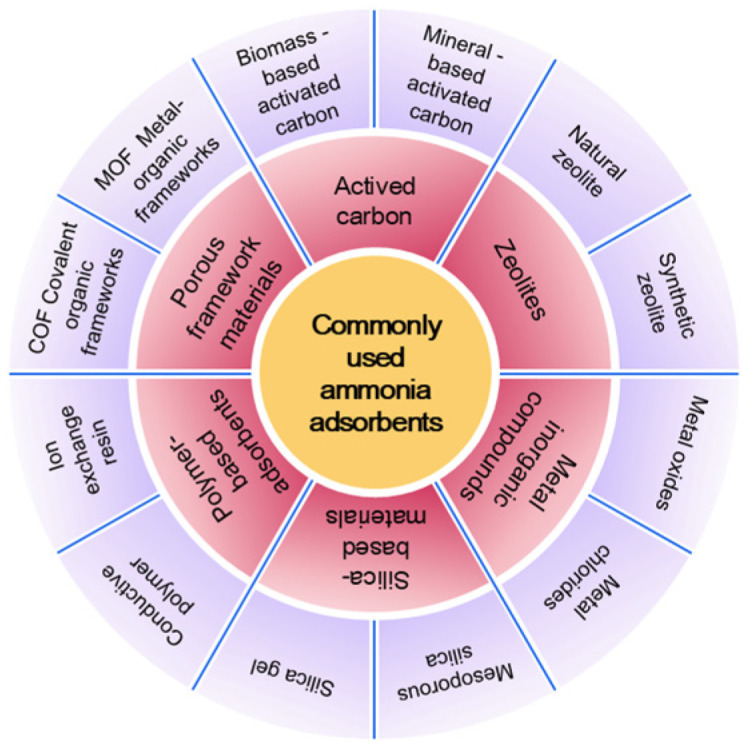
Common types of ammonia adsorbents.

The adsorption method has emerged as a predominant technology for ammonia pollution control due to its operational flexibility, low energy consumption, and recyclability. Currently, widely used ammonia adsorbents include AC, as well as zeolite molecular sieves and similar materials, which can achieve the selective capture of ammonia molecules through mechanisms such as physical adsorption, chemical coordination, and acid–base neutralization (Figure 2). In this section, we systematically review extensively used ammonia adsorption materials for industrial applications. We subsequently critically analyze their core advantages and limitations, with the aim of providing scientific insights for material optimization and scenario-specific application strategies.

### 3.1. Activated Carbon

AC is mainly composed of C, H, O, and N, along with other elements. Due to its large specific surface area and rich pore structure, AC exhibits excellent adsorption performance for many pollutants, especially aromatic compounds such as benzene and toluene. AC can also be modified through methods such as washing, pickling, and alkali washing, according to the need to adsorb different types of impurities [26].

Huang et al. [27] determined the influence of the surface acidity of AC on the adsorption effect of ammonia gas through different modifications. The ammonia adsorption capacities of modified AC could be improved, with nitric acid-modified AC achieving the highest rate of ammonia penetration with 2.493 mmol g^−1^, which was significantly higher than that achieved by unmodified AC (0.134 mmol g^−1^). Qajar et al. [28] obtained a series of microporous carbons by oxidizing alcohols with CO_2_. Using the same nitric acid treatment method, the static adsorption capacity of NH_3_ increased from an initial 10 mmol g^−1^ to 17 mmol g^−1^. The strong acid–base interaction between ammonia and the surface functional groups of AC was found to be the main reason for the increased ammonia adsorption capacity.

Until now, a significant amount of AC, especially modified AC, has been used for gas adsorption and other needs, despite it exhibiting poor adsorption activity for ammonia and carrying the risk of braising under partial use conditions. These are issues that must be addressed in future research on AC materials.

### 3.2. Graphene Oxide

Unlike AC, graphene oxide (GO) consists of a honeycomb flat film, formed by means of the hybridization of carbon atoms with sp^2^. As a product of graphene stripping, GO has a large specific surface area and excellent two-dimensional structure. Zhang et al. [29] demonstrated through density functional theory (DFT) calculations that water molecules on a graphene surface in a humid environment promoted the conversion of NH_3_ to NH_4_^+^, resulting in a significant increase in adsorption energy from 31 to 45 meV (for pure NH_3_ on graphene under dry conditions) to 528–644 meV. Seredych et al. [30] prepared graphene by placing graphite powder in concentrated sulfuric acid with potassium permanganate, followed by calcination. The adsorption capacity of uncalcined graphene reached 3.59 mmol g^−1^. Moreover, the NH_3_ adsorption capacity of the GO decreased with the decrease in surface acidity after washing, indicating that the water vapor in the NH_3_ gas promoted the dissociation of carboxyl groups and interaction with NH_3_. However, the performance of graphene for ammonia adsorption under different humidity levels varies significantly, leading to a limited application scenario.

### 3.3. Zeolite

Zeolite is a crystalline material with a tetrahedral framework composed of silicon and aluminum with a highly porous structure. These structural characteristics give zeolites excellent ion exchange properties, adsorption selectivity, and thermal stability [31]. Bernal et al. [32] measured the adsorption effect of four different natural zeolites for NH_3_ and reported a maximum dynamic adsorption capacity of 0.831 mol g^−1^. In addition to natural zeolites, molecular sieves made from artificially modified zeolite can improve the properties of zeolites by adjusting the pore structure. Helminen et al. [33] found that the adsorption capacity of directionally synthesized 13X-zeolite reached 9.326 mmol g^−1^ at 93.8 kPa, which is higher than that of natural zeolites (5.904 mmol g^−1^). This was attributed to the increased surface activity of the zeolite materials. Witter et al. [34] found that zeolites could adsorb NH_3_ more effectively at lower pressures. However, unlike AC, the adsorption performance of zeolites for NH_3_ significantly decreased in the presence of water vapor. This was mainly because the presence of water molecules clogged the internal pores of the zeolites, leading to a decrease in their adsorption capacity and adsorption rate.

### 3.4. Metal Inorganic Compounds

Most metal inorganic compounds can react with ammonia through complexation reactions, forming coordination compounds, with notable examples including activated alumina and CaCl_2_ [35]. Helminen et al. [33] measured the ammonia adsorption capacities of three types of aluminum oxide adsorbents, Alumina VPO_2_, Alumina 1593, and Alumina 1597, and found that their adsorption capacities at 298.15 K and 100 kPa were 2.606, 2.159, and 3.008 mmol g^−1^, respectively. Kim et al. [36] synthesized mesoporous aluminum oxide BSMA using Al(NO_3_)_3_·9H_2_O as the alumina precursor, achieving an ammonia adsorption capacity of 3.77 mmol g^−1^. During the adsorption process, the ammonia first adsorbed on the outer surface of the BSMA and then adsorbed into the inner pores of the material. In addition, with a decreasing particle size, the adsorption performance increased, with an increasing number of effective sites.

Although metal inorganic compounds have good adsorption capacity for NH_3_, their slow adsorption kinetics, significant volume expansion, and difficult desorption limit their application [37,38]. Therefore, instead of directly using metal inorganic compounds as ammonia adsorbents, researchers have preferred to incorporate them into other materials as loading materials to enhance their ammonia adsorption performance.

In summary, these materials exhibit distinct advantages and limitations: activated carbon demonstrates a rapid adsorption capability owing to its high specific surface area and rich porous structure; however, it shows a low adsorption capacity for ammonia, meaning that it requires surface modification. Graphene oxide achieves strong chemisorption with ammonia through oxygen-containing functional groups, yet its practical performance is hindered by interlayer stacking. Zeolites achieve high adsorption selectivity due to their ordered microporous structure and ion exchange properties, but water will compete with ammonia, resulting in a reduced adsorption capacity in high-humidity environments. Although metal inorganic compounds have a high adsorption capacity and thermal stability, the difficulty of desorption and the volume expansion during the adsorption process significantly limit its large-scale utilization. Hence, researchers have developed novel ammonia adsorption materials to enhance ammonia adsorption capabilities.

## 4. PFMs in Ammonia Adsorption

In recent years, PFMs, including covalent organic frameworks (COFs), hydrogen-bonded organic frameworks (HOFs), and metal–organic frameworks (MOFs), have emerged as promising materials for ammonia adsorption due to their high surface area, tunable pore structure, and excellent adsorption properties. COFs are an emerging class of crystalline porous materials that are composed of light elements (e.g., H, B, C, N, and O) which are connected via covalent bonds. Using state-of-the-art molecular design, COFs can exhibit a large surface area, unique porosity, high crystallinity, and tunable pore chemistry, with promising applications in the fields of catalysis, sensing, and gas adsorption [39,40,41]. HOFs are formed entirely from the self-assembly of weaker hydrogen bonds among organic molecules. The flexibility and reversibility of hydrogen bonds make it easy for HOFs to return to their initial state when the framework collapses after a stimulus response [42,43,44]. MOFs are formed through the coordination of transition metal ions with organic ligands. As a class of promising solid adsorbents, MOFs have received significant attention in gas separation due to their regular nanopores, adjustable porosity, high specific surface area, and easy functionalization [45,46,47,48]. Through chemical modification, pore engineering, and functional design, these materials can achieve high selectivity and the efficient storage of ammonia. For example, COF materials, with a highly ordered pore structure and chemical stability, have demonstrated excellent ammonia adsorption performance [49]. Furthermore, by introducing open metal sites or acidic functional groups, certain MOF materials can significantly enhance the interaction with ammonia, thereby improving their adsorption capacity [50]. In this section, we review the latest research progress in the field of ammonia adsorption using porous framework materials, focusing on their adsorption mechanisms, performance optimization strategies, and potential applications.

### 4.1. COFs and HOFs

**Figure 3:**
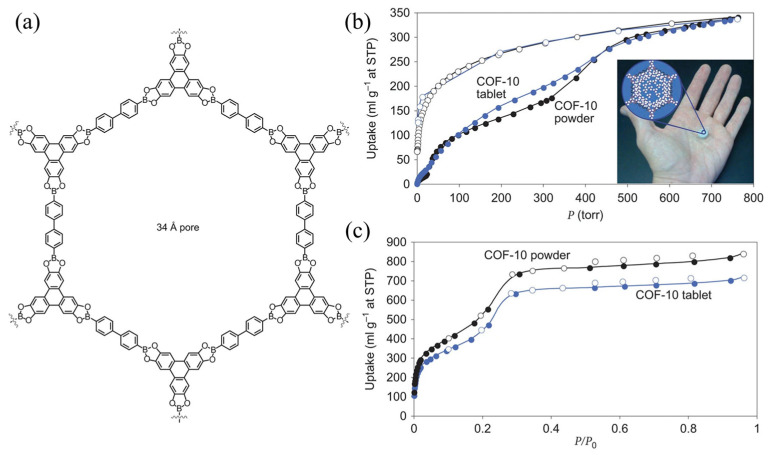
(**a**) Schematic representation of COF-10; (**b**) ammonia uptake at 298 K for COF-10 and COF-10 tablets and pressed tablets of COF-10 loaded with ammonia; and (**c**) nitrogen gas adsorption isotherms (77 K), tested on the same COF-10 powder and COF-10 tablets (Solid symbols represent adsorption, hollow symbols represent desorption) [51].

Many COFs contain high-density Lewis acid sites that can interact with ammonia, resulting in excellent ammonia adsorption and storage. Doonan et al. [51] evaluated the ammonia adsorption effects of COF-10 and demonstrated that the static ammonia adsorption capacity of COF-10 at 298 K and 101 kPa was 15 mmol g^−1^, which was higher than those of other porous materials. Meanwhile, desorption was completed at 473.15 K under a vacuum environment. Because the material mainly adsorbed NH_3_ through Lewis acid–base interactions, it maintained a stable skeleton structure after multiple adsorption–desorption cycles (Figure 3).

**Figure 4:**
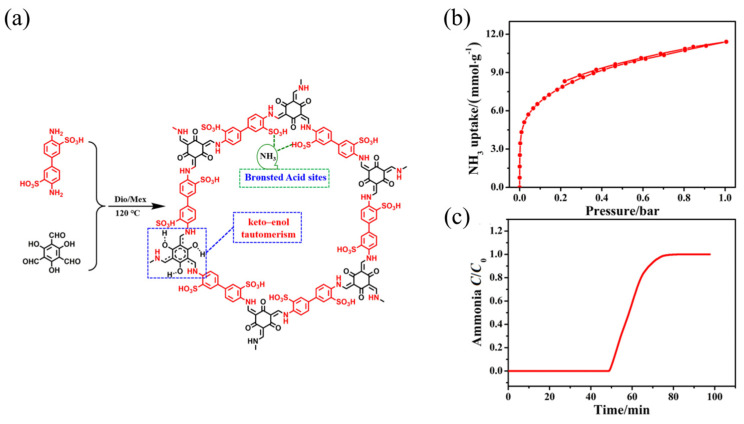
(**a**) Synthesis and structure of TpBD-(SO_3_H)_2_; (**b**) NH_3_ adsorption isotherm of TpBD-(SO_3_H)_2_; and (**c**) NH_3_ breakthrough curve of TpBD-(SO_3_H)_2_ at 298 K [52].

Li et al. [52] synthesized a highly stable sulfonic acid COF using triformylphloroglucinol and diaminobenzene disulfuric acid. This COF retained its structural integrity after immersion in water, acid, and organic solvents for 48 h due to the covalent bonds that formed from the condensation of aldehyde and amine groups. The strong interaction between the sulfonic acid sites and ammonia molecules resulted in an ammonia adsorption capacity of 11.5 mmol g^−1^ at 298 K (Figure 4).

**Figure 5:**
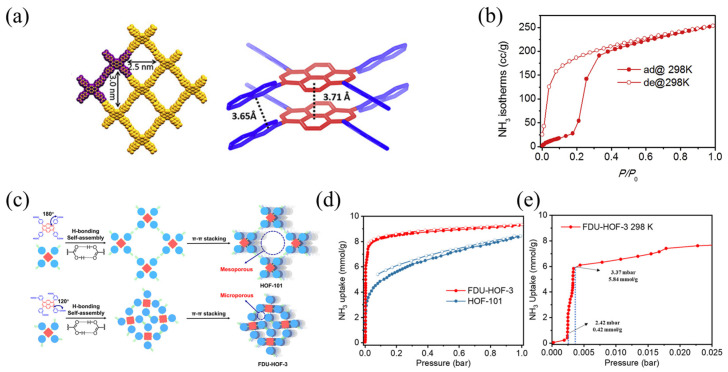
(**a**) Crystal structures of HOF-102; (**b**) ammonia adsorption isotherm of HOF-102 at 298 K; (**c**) schematic of H-bonding self-assembly and π−π stacking in HOF-101 and FDU-HOF-3; (**d**) NH_3_ adsorption and desorption isotherms of HOF-101 and FDU-HOF-3 at 298 K; and (**e**) adsorption capacity of FDU-HOF-3 under low ammonia concentrations at 298 K (Solid symbols represent adsorption, hollow symbols represent desorption) [37,53].

Ma et al. [53] measured the ammonia adsorption capacity of HOF-102 to be 250 cm^3^ g^−1^ at 298 K and 1 atm (Figure 5). In addition, Song et al. [37] synthesized two carboxylic acids, HOF-101 and FDU-HOF-3, with an adaptive NH_3_ adsorption capacity and phase transition (Figure 5). The adsorption capacity of FDU-HOF-3 reached 8.13 mmol g^−1^ at 25 mbar, while FDU-HOF-3 exhibited significant potential for low-concentration ammonia capture and high-concentration ammonia storage.

Although COFs and HOFs differ in their structural construction, their widespread practical application remains limited by high synthesis costs, and addressing this challenge is crucial for commercialization (Table 2). In addition, their thermal and chemical stability must be improved. Choosing both simple and easily obtained monomers and enhancing the thermochemical stability are the main future research directions.

### 4.2. MOF Materials

As a strong Lewis acid and Brønsted base, ammonia can react with most metal ions through coordination. As a result, many metal salts have a high theoretical ammonia adsorption capacity. However, the direct use of metal salts poses challenges, such as slow adsorption kinetics, volume expansions during adsorption, and difficult desorption [37,38]. Each metal unit in MOFs serves as a potential adsorption site for NH_3_; therefore, compared with metal salts, MOFs offer a superior choice for ammonia adsorption [45].

In 2008, Yaghi et al. [56] evaluated the ammonia adsorption performance of five MOFs, including MOF-5 and MOF-199. The study, which compared these MOFs to Calgon BPL activated carbon, revealed that the porosity of MOFs played a decisive role in their adsorption capacity, with MOF-199 exhibiting the highest dynamic ammonia adsorption capacity of 87 mg g^−1^, marking the first complete report of ammonia adsorption by MOFs.

**Figure 6:**
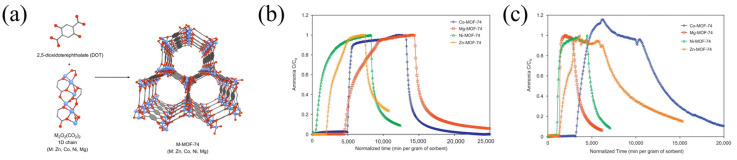
(**a**) Structure of MOF-74; (**b**) ammonia breakthrough curves of MOF-74 under dry (RH = 0%) conditions; and (**c**) ammonia breakthrough curves under humid (RH = 80%) conditions [57].

Glover et al. [57] synthesized MOF-74 with four different metal centers, namely cobalt, magnesium, nickel, and zinc (Figure 6), and measured their ammonia adsorption properties. The study found that different metal centers significantly affected the adsorption results. Under the same drying conditions, Mg-MOF-74 reached the highest capacity of 7.6 mmol g^−1^, while Ni-MOF-74 had the lowest of only 2.3 mmol g^−1^. The experimental results revealed that the superior performances of Co-MOF-74 and Mg-MOF-74 were due to their coordinatively unsaturated sites. Desorption experiments demonstrated that Co-MOF-74 and Mg-MOF-74 retained 70% and 83% of the adsorbed ammonia, respectively, whereas Ni-MOF-74 and Zn-MOF-74 retained 51.3% and 58.6% after desorption under dry conditions.

**Figure 7:**
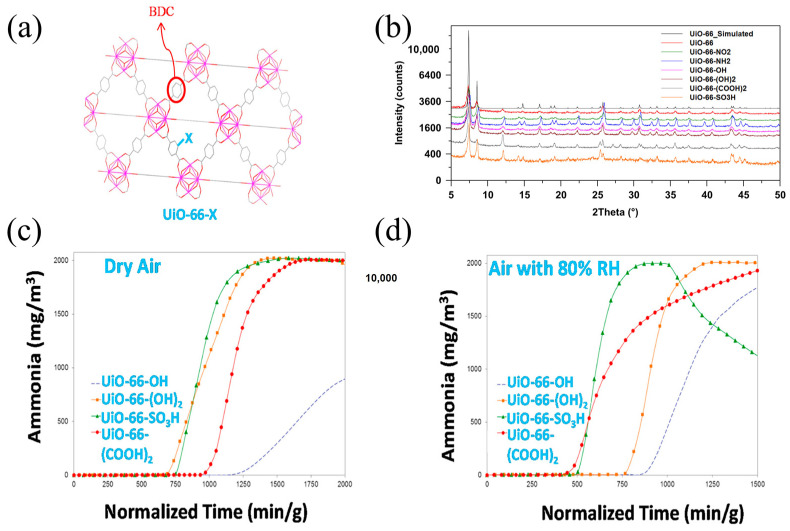
(**a**) An illustration of the UiO-66-X framework structure; (**b**) PXRD patterns of as-synthesized UiO-66 and UiO-66-X variants; (**c**) NH_3_ breakthrough curves under dry air conditions; and (**d**) NH_3_ breakthrough curves under 80% RH air conditions (Solid symbols represent adsorption, hollow symbols represent desorption) [58].

Walton et al. [58] investigated the ammonia removal abilities of UiO-66 and its isomorphism and successfully synthesized six isomers of UiO-66 by changing the introduced ligands. Their results showed that the ammonia adsorption capacities of UiO-66-SO_3_H and UiO-66-(COOH)_2_ were lower than those of UiO-66-OH and UiO-66-NH_2_. UiO-66-OH had the highest NH_3_ adsorption capacity of 5.69 mmol g^−1^ under dry conditions (Figure 7) because the incorporation of larger functional groups such as carboxyl groups caused a decrease in material porosity. However, under humid conditions, the ammonia adsorption capacity of functionalized UiO-66 significantly decreased due to the competitive adsorption of water and ammonia molecules, with the highest adsorption capacity being only 5.69 mmol g^−1^.

Chen et al. [59] synthesized M-2(INA) (M = Cu, Co, Ni, and Cd) materials through dehydration with M(INA)_2_(H_2_O)_4_ and demonstrated that M-2(INA) could be reversibly converted to M(INA)_2_(H_2_O)_2_(NH_3_)_2_ under wet conditions. The dry NH_3_ adsorption capacity reached 12–13 mmol g^−1^, while the adsorption capacity under wet conditions and the same temperature conditions was 5–6 mmol g^−1^.

**Figure 8:**
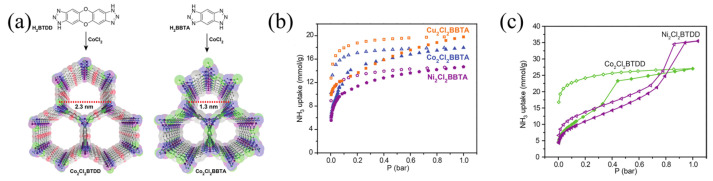
(**a**) Synthesis and structure of Co_2_Cl_2_BTDD and Co_2_Cl_2_BBTA; (**b**) NH_3_ adsorption and desorption of Co_2_Cl_2_BBTA, Ni_2_Cl_2_BBTA, and Cu_2_Cl_2_BBTA; and (**c**) NH_3_ adsorption and desorption of Co_2_Cl_2_BTDD and Ni_2_Cl_2_BTDD at 298 K (Solid symbols represent adsorption, hollow symbols represent desorption) [60].

Rieth et al. [60] synthesized several microporous triazolate metal–organic frameworks containing open metal sites. The static ammonia adsorption capacities of CoCl_2_BBTA, NiCl_2_BBTA, and CuCl_2_BBTA (Figure 8) were 17.95, 14.68, and 19.79 mmol g^−1^, respectively. Although the adsorption capacity of BTDD reached 35 mmol g^−1^ at 263 K, due to the presence of more metal sites, BBTA with small pores exhibits a higher adsorption capacity than BTDD with large pores.

Chen et al. [61] evaluated the NH_3_ adsorption capacities of HKUST-1, MIL-100(Fe), and UiO-66 under low-pressure conditions, with the results showing that their static ammonia adsorption capacities were 17.73, 8.54, and 8.51 mmol g^−1^, respectively, at 0–0.13 *P/P*_0_. These findings were in agreement with the calculated results of HKUST-1 (−147.89 kJ mol^−1^) > MIL-100(Fe) (−80.28 kJ mol^−1^) > UiO-66 (−47.08 kJ mol^−1^).

**Figure 9:**
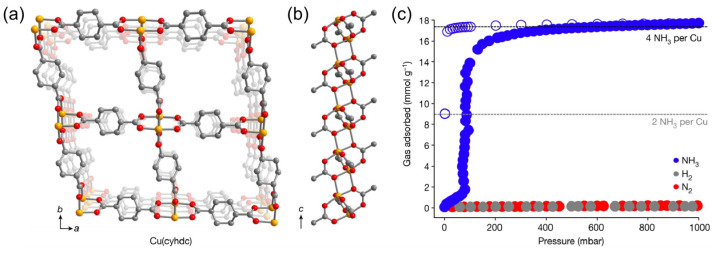
(**a**) Illustration of the Cu(cyhdc) framework structure; (**b**) side view of the one-dimensional copper paddlewheel chain motif defining the pore vertices; and (**c**) NH_3_ adsorption and desorption isotherms at 298 K for Cu(cyhdc) [62].

Synder et al. [62] reported an air-stable and crystalline framework, Cu(cyhdc) (cyhdc^2−^ = trans1,4-cyclohexanedicarboxylate), with a saturated coordination Cu(II) site. Due to the reversible temperature- and pressure-dependent co-phase transition of NH_3_ on the material, Cu(cyhdc) could adsorb NH_3_ more selectively than N_2_ or H_2_. At 298 K, about 16 mmol g^−1^ was gradually absorbed at 80 mbar, reaching a plateau above 100 mbar. At 1 bar, the maximum adsorption capacity was 17.5 mmol g^−1^, corresponding to the adsorption of four equivalents of NH_3_ per Cu site (Figure 9). During desorption, a significant lag was observed in the Cu(NH_3_)_4_(cyhdc) isotherm, and only half of the NH_3_ was desorbed at 1 mbar, indicating that intermediates were present, with two NH_3_ per Cu during desorption, and blue Cu(NH_3_)_4_(cyhdc) transformed into purple during desorption. A single-crystal X-ray diffraction analysis confirmed the formation of a one-dimensional Cu(NH_3_)_2_(cyhdc) solid, where two trans-NH_3_ and two trans-carboxylic acid ligands coordinated through a single oxygen atom in the Cu(II) central coordination of each square plane. Meanwhile, at lower pressures, the desorption plateaued at one ammonia per Cu, which could be attributed to the formation of Cu(NH_3_)(cyhdc).

Han et al. [63] synthesized a robust MOF structure, MFM-300(M) (M = Fe, V, Cr, and In), for NH_3_ adsorption, which had a high absorption rate of 17.3 mmol g^−1^ and could maintain an adsorption capacity of 16.1 mmol g^−1^ after 20 cycles at 273 K and 1 bar. In addition, MFM-300(Cr^VIII^) demonstrated better NH_3_ adsorption stability in wet environments than MFM-300(Fe^VIII^), indicating that even the saturation coordination of the metal center significantly affected the stability of the MOF in the presence of guest molecules.

The ammonia adsorption abilities of numerous MOFs have been evaluated (Table 3). Although the feasibility of MOFs for ammonia adsorption has been demonstrated experimentally, ensuring their commercialization or industrialization remains challenging, and the stability, reversibility, durability, and production cost of these materials cannot be ignored. Moreover, MOFs with a high NH_3_ adsorption capacity are generally composed of Cu^2+^ and Zn^2+^ nodes, which are prone to structural collapse during adsorption due to the strong coordination between metal nodes and NH_3_ [37,38]. Many high-capacity MOFs also exhibit reduced adsorption performance under varying environmental conditions, limiting their practical applicability. Therefore, rather than relying solely on MOFs, the development of functional MOF composite materials presents a promising alternative for improving their stability and performance under real-world conditions.

### 4.3. PFM Composite Materials

The combination of porous structural materials with chemical adsorbents has been shown to enhance both mass transfer and ammonia adsorption properties [66]. Inspired by these strategies, novel PFM composites can be designed to enhance the adsorption capacity of traditional PFMs. Approaches for this include functionalization with acidic groups that impart moderate hydrophobicity, anchoring halides or metal aggregates, and adjusting the pore size relative to the critical diameter.

Yang et al. [55] synthesized [CaOOC]_17_-COF, [MnOOC]_17_-COF, and [SrOOC]_17_-COF by immersing the COF in CaCl_2_, MnCl_2_, and SrCl_2_ solutions. They then anchored metals into the COF through coordination interactions to obtain composite materials. After modification, the [SrOOC]_17_-COF with the highest adsorption capacity achieved 14.3 mmol g^−1^ at 298 K, significantly surpassing that of the unmodified [HOOC]_17_-COF (6.85 mmol g^−1^). This novel approach enables the development of tailor-made porous materials with tunable pore-engineered surfaces for ammonia uptake.

Petit et al. [67] synthesized composites of MOF-5 and GO at different proportions and tested the adsorption capacity of ammonia under dry conditions. The dynamic adsorption capacity of the composite was increased to 82 mg g^−1^ at 1000 ppm and 450 mL min^−1^ due to its high porosity; however, excessive ammonia adsorption eventually led to structural collapse. More et al. [68] adopted a similar strategy by introducing partially reduced graphene oxide (rGO) into Zn-BDC and optimizing it for NH_3_ sensing at low concentrations, with a reaction time of 60/120 s at 20 ppm.

**Figure 10:**
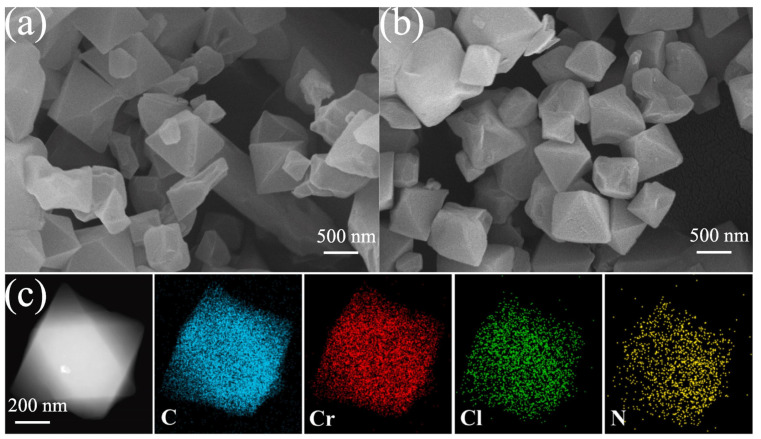
(**a**) SEM images of MIL-101(Cr); (**b**) SEM images of [CAM][Cl]@MIL-101(Cr)-30%; and (**c**) TEM images and elemental mapping of [CAM][Cl]@MIL-101(Cr)-30% [69].

Han et al. [69] synthesized a composite material, [CAM][Cl]@MIL-101(Cr), based on MIL-101(Cr) and ionic liquids (ILs). According to their scanning electron microscopy (SEM) results, the ILs deposited irregular translucent layers on the MIL-101(Cr) surface, while no translucent layer was observed using transmission electron microscopy (TEM). The uniform distribution of Cl and N in the material, revealed by means of EDS, indicated that the ILs were uniformly distributed on the micron scale (Figure 10), and the saturated adsorption capacity of [CAM][Cl]@MIL-101(Cr)-30% reached 11.61 mmol g^−1^, which was much higher than the 8.03 mmol g^−1^ of the pristine MIL-101(Cr). The separation selectivity for NH_3_/CO_2_ (S_NH_3_/CO_2__) of the composite was 3266, which was 3.13 times higher than that of MIL-101 (Cr).

**Figure 11:**
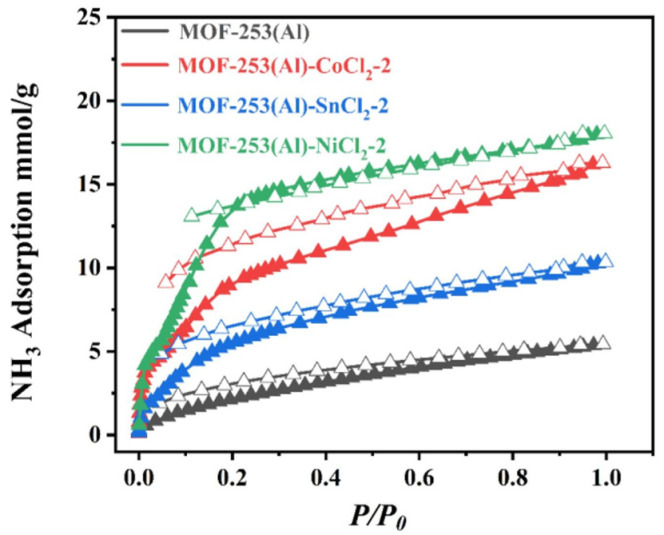
NH_3_ adsorption isotherm of MOF-253(Al) and three functionalized MOFs (Solid symbols represent adsorption, hollow symbols represent desorption) [70].

Wang et al. [70] designed and synthesized a composite material by anchoring three metal chlorides (NiCl_2_, CoCl_2_, and SnCl_2_) to the bipyridine group in MOF-253(Al). The highest NH_3_ absorption rate was that of MOF-253(Al)-NiCl_2_-2 at 18 mmol g^−1^, which was 3.33 times that of the original MOF-253(Al) (5.5 mmol g^−1^), and the adsorption kinetics were significantly faster than those of the original MOF-253(Al) (Figure 11). In addition, the material demonstrated excellent NH_3_ selectivity, with S_NH_3_/N_2__ and S_NH_3_/H_2__ coefficients of 5708 and 2320, respectively. According to the infrared spectroscopy, X-ray photoelectron spectroscopy (XPS), and DFT studies, this excellent absorption and selectivity was attributed to the coordination of the nitrogen atom of NH_3_ with Ni^2+^ and the synergistic interaction of the hydrogen bonds of NH_3_ with guest Cl^−^ and carboxyl O atoms on the MOF.

Li et al. [48] impregnated ionic liquids in Al-fum, and the ammonia adsorption capacity results revealed that the capacities of [C_2_N][Zn_3_Cl_7_]@Al-fum-60% at 80 °C and 100 °C were 9.9 and 8.8 mmol g^−1^, respectively. Therefore, using the coordination interaction between ammonia and the ionic liquid Zn center is a promising method for promoting ammonia adsorption.

PFM composites, engineered by integrating PFMs with functional materials, address the inherent limitations of pristine PFMs and exhibit synergistically enhanced adsorption capacity, catalytic activity, and stability. However, current challenges lie in the poor interfacial compatibility and inhomogeneity of composites, which may obscure active sites or increase the mass transfer resistance. Additionally, complex fabrication processes often elevate costs and hinder scalable production, while the long-term cyclability of certain composite systems remains inadequately validated. Future research should prioritize interface engineering and sustainable synthesis strategies to reconcile performance enhancement with practical applicability.

## 5. Conclusions and Future Perspectives

In this review, we introduced several key NH_3_ capture techniques, focusing on physisorption and chemisorption for ammonia removal. We also compared the research progress, adsorption capacity, and advantages and disadvantages of materials such as AC, zeolite molecular sieves, COFs, and MOFs. With tunable porosity, a high specific surface area, and the ability to incorporate various functional groups, PFMs offer multiple mechanisms for ammonia adsorption, including physical and chemical adsorption and hydrogen bonding, making them highly promising materials for efficient NH_3_ capture. Moreover, their well-defined channels allow for precise structural modifications to enhance their adsorption capacity. These methods include increasing the number of acidic functional groups to expose more acidic sites, loading efficient adsorbents, and substitutions with more suitable metal groups and organic ligands.

Despite these advantages, PFMs face significant challenges in ammonia adsorption/desorption, including high production costs, lengthy preparation cycles, and poor stability and durability. Although these materials exhibit excellent ammonia adsorption performance under ambient temperature and pressure conditions, performance degradation in high-humidity environments remains a critical issue. In addition, the ammonia adsorption mechanisms of some PFMs remain poorly understood, and explorations of their microscopic interaction mechanisms, as well as an explanation of their structural collapse, remain lacking, limiting the further development of PFMs for ammonia adsorption. Future research should explore the structural optimization and functional enhancement of PFMs further to improve their ammonia adsorption performance in complex environments and promote their practical applications in industrial waste gas treatment, ammonia storage, and separation. We anticipate the development of highly efficient, stable, and easily synthesized PFMs that can adapt to real-world conditions, along with innovative design strategies to maximize their impact in environmental remediation and related fields.

## Figures and Tables

**Table 1:** Advantages and disadvantages of various industrial ammonia abatement techniques.

Method	Advantages	Disadvantages
Catalytic oxidation	Simple Stable	High energy requirements The generation of NO_x_
Membrane separation	Low maintenance requirements Easy to install and operate	Low stability Low selectivity under low flux
Biotechnology	Low pressure drops Low operating costs	Complex operation Bacteria release
Washing	Large temperature ranges Sturdy scrubbers	Corrosion Furring
Adsorption	Low cost Operatable under ambient conditions High selectivity Recycling	Limited capacity High regeneration energy

**Table 2:** Static adsorption capacities and specific surface areas of different types of COF and HOF materials.

COF/HOF	BET Surface Area (m^2^ g^−1^)	Adsorption Capacity (mmol g^−1^)	Temperature and Pressure	Ref.
HOF-101	2100	8.44	298 K, 1 atm	[37]
FDU-HOF-3	310	9.34	298 K, 1 atm	
COF-10	1200	15	298 K, 1 atm	[51]
TpBD-(SO_3_H)_2_	-	11.5	298 K, 1 atm	[52]
HOF-102	2500	11.16	298 K, 1 atm	[53]
KUF-1a	-	6.67	283 K, 1 atm	[54]
[HOOC]_17_-COF	652	6.85	298 K, 1 atm	[55]
[HOOC]_33_-COF	458	8.21	298 K,1 atm	

- Not mentioned in the reference.

**Table 3:** Static adsorption capacities and specific surfaces area of different types of MOF materials.

MOF	BET Surface Area (m^2^ g^−1^)	Adsorption Capacity (mmol g^−1^)	Temperature and Pressure	Ref.
MOF-5	2449	12.2	298 K, 1.05 atm	[56]
MOF-177	3275	12.2	298 K, 1.05 atm	
HKUST-1	909	6.8	298 K, 1 atm	
Cu-MOF-74	1170	3.4	298 K, 1 atm	[57]
Co-MOF-74	825	6.7	298 K, 1 atm	
Mg-MOF-74	1206	7.6	298 K, 1 atm	
UiO-66	1100	1.79	273 K, 1 atm	[58]
UiO-66-NH_2_	1096	3.56	273 K, 1 atm	
UiO-66-NO_2_	729	1.98	273 K, 1 atm	
UiO-66-OH	946	5.69	273 K, 1 atm	
UiO-66-(OH)_2_	814	2.29	273 K, 1 atm	
UiO-66-SO_3_H	323	2.24-	273 K, 1 atm	
UiO-66-(COOH)_2_	221	2.83	273 K, 1 atm	
CoCl_2_BTDD	-	4.78	298 K, 1 atm	[60]
CoCl_2_BBTA	1106	8.56	298 K, 1 atm	
CuCl_2_BBTA	1205	7.52	298 K, 1 atm	
Cu(cyhdc)	490	17.5	298 K, 1 atm	[62]
MFM-300(V^IV^)	1565	17.3	273 K, 1 atm	[63]
MFM-300(Fe)	1192	16.1	273 K, 1 atm	
MFM-300(V^III^)	1892	15.6	273 K, 1 atm	
MFM-300(Cr)	1045	14.0	273 K, 1 atm	
NFU-4	-	17.7	298 K,1 atm	[64]
NU-300	1470	8.41	298 K,1 atm	[65]

- Not mentioned in the reference.

## Abbreviations

PFMs	Porous framework materials
MOFs	Metal–organic frameworks
COFs	Covalent organic frameworks
HOFs	Hydrogen-bonded organic frameworks
AC	Activated carbon
GO	Graphene oxide
